# Supplementary material for: Empowering a qudit-based quantum processor by traversing the dual bosonic ladder
Source: Nat Commun. 2024 Aug 19;15:7117. doi: 10.1038/s41467-024-51434-2 (PMC11333499; doi:10.1038/s41467-024-51434-2)
Supplement: Supplementary file 1 — Supplementary Information [file 41467_2024_51434_MOESM1_ESM.pdf]

Supplementary Information for "Empowering a qudit-based quantum processor by traversing the dual bosonic ladder"

Supplementary Note 1 – Experimental Device

Device description

The experiment is performed on a superconducting quantum processor consisting of eight connected transmon circuits forming a ring, where each transmon serves as the qudit. A false-colour image of a representative device is displayed in Supplementary Figure 1(**a**). The capacitive energy is designed to be $E_C \sim 270$ MHz, and the Josephson energy is used to tune the qudit's $0-1$ frequency to be around 5.5 GHz. The qudits are pairwise connected using resonator couplers. Each qudit is capacitively connected to a separate microwave driving line and dispersively coupled to a readout resonator. The frequency allocation of the qudits and readout resonator used in the experiments is shown in Supplementary Figure 1(**b**). The resonator couplers are designed to have their resonant frequencies at approximately 7.2 GHz and qubit-resonator coupling strength of about 75 MHz. The effective qudit-qudit subspace coupling can be approximated as $J \approx g_{1r}g_{2r}\left(\Delta_{1r}^{-1}+\Delta_{2r}^{-1}\right)/2$, where g_{ir} is the direct coupling coefficient between qudit i and the resonator, ω_r is the frequency of the resonator, and $\Delta_{ir}=\tilde{\omega}_{q,i}-\omega_r$ is the qudit-resonator detuning. Here, we define $\tilde{\omega}_{q,i}=\omega_{q,i}+g_{ir}^2/(\omega_{q,i}-\omega_r)$, where $\tilde{\omega}_{q,i}$ ($\omega_{q,i}$) is the relevant dressed (bare) transition frequency of qudit i. This results in a coupling coefficient $J_{01}/2\pi \sim 3$ MHz in the $0-1$ subspace.

Parameters	Q_1	Q_2	Q_3	Q_4
$\omega_{01}/2\pi$ (GHz)	5.333	5.396	5.572	5.745
$\omega_{12}/2\pi$ (GHz)	5.061	5.124	5.303	5.465
$\omega_{23}/2\pi$ (GHz)	4.757	4.821	5.005	5.171
$\omega_r/2\pi$ (GHz)	6.563	6.622	6.678	6.736
$\kappa/2\pi$ (MHz)	1.44	1.45	1.83	1.73
$\chi_{01}/2\pi$ (MHz)	1.09	1.09	1.26	1.44
Avg. T_1^{01} (μs)	50(4)	49(4)	60(5)	61(9)
Avg. T_1^{12} (μs)	35(2)	35(4)	31(8)	34(2)
Avg. T_1^{23} (μs)	24(4)	26(3)	23(4)	15(1)
Avg. T_{2e}^{01} (μs)	78(5)	85(9)	90(6)	74(5)
Avg. T_{2e}^{12} (μs)	57(4)	57(4)	56(9)	34(2)
Avg. T_{2e}^{23} (μs)	26(2)	27(2)	24(3)	20(2)
$J_{01}/2\pi$ (MHz)	2.5	2.5	-	-
	-	2.7	2.7	-
	-	-	3.1	3.1

Supplementary Table 1. Qudit parameters of the experimental device. Here, g is the coupling strength between the qudits and their respective readout resonators, and J_{01} refers to the effective coupling strength between the qudits' $0-1$ subspace. The coherence statistics (average values and standard deviations, see Supplementary Figure 1**c**) are each obtained from an ensemble of 100 measurements.

Device fabrication

The device was fabricated on a high-resistivity silicon wafer ($\rho > 10$kΩ-cm) with Niobium (Nb) and Aluminum (Al). After cleaning the wafer with piranha (mixture of sulfuric acid and hydrogen peroxide heated to 120°C) and hydrofluoric acid (HF) to remove the organics and silicon oxide, a 200-nm layer of Nb was sputtered. Then, the superconducting circuit components except the junctions are defined by the photo-lithography technique and reactive-ion etching. We intentionally over-etched the silicon substrate to avoid short circuits and reduce the dielectric loss.

The wafer was then cleaned with buffered oxide etch (BOE) solvent before the fabrication of Josephson junctions. Josephson junctions are defined by e-beam lithography and deposited by triple-angle e-beam evaporation of Al films following the Manhattan style technique. A gentle plasma cleaning is applied before the Al film deposition to clean the e-beam resist residues after the development and enhance the bond between the substrate and the Al films. The galvanic contact between the Josephson junctions and their capacitor pads was formed by Ar ion-milling band-aid process.

Finally, the fabricated wafer was coated with methyl methacrylate (MMA) resist to protect it before dicing. After dicing, the chips are cleaned with N-methylpyrrolidone (NMP) at 80°C and packaged in a copper box for testing in the dilute refrigerators. More fabrication details can be found in Ref. [1].

Single-qudit control

The transmon Hamiltonian reads

$$\hat{H}/h = 4E_C(\hat{n}-n_g) - E_J\cos\hat{\varphi}, \tag{1}$$

where $\hat{n}$ and $\hat{\varphi}$ are respectively the Cooper-pair number operator and gauge-invariant phase operator. For energy ratio $E_J/E_C > 50$, the system admits many bound states that can be spectrally resolved and coherently addressed. One can approximate the Hamiltonian of the transmon as that of a Duffing Oscillator with frequency $\omega_{01}/2\pi \approx \sqrt{8E_JE_C}-E_C$ and self-Kerr $\alpha/2\pi \approx E_C$. By introducing

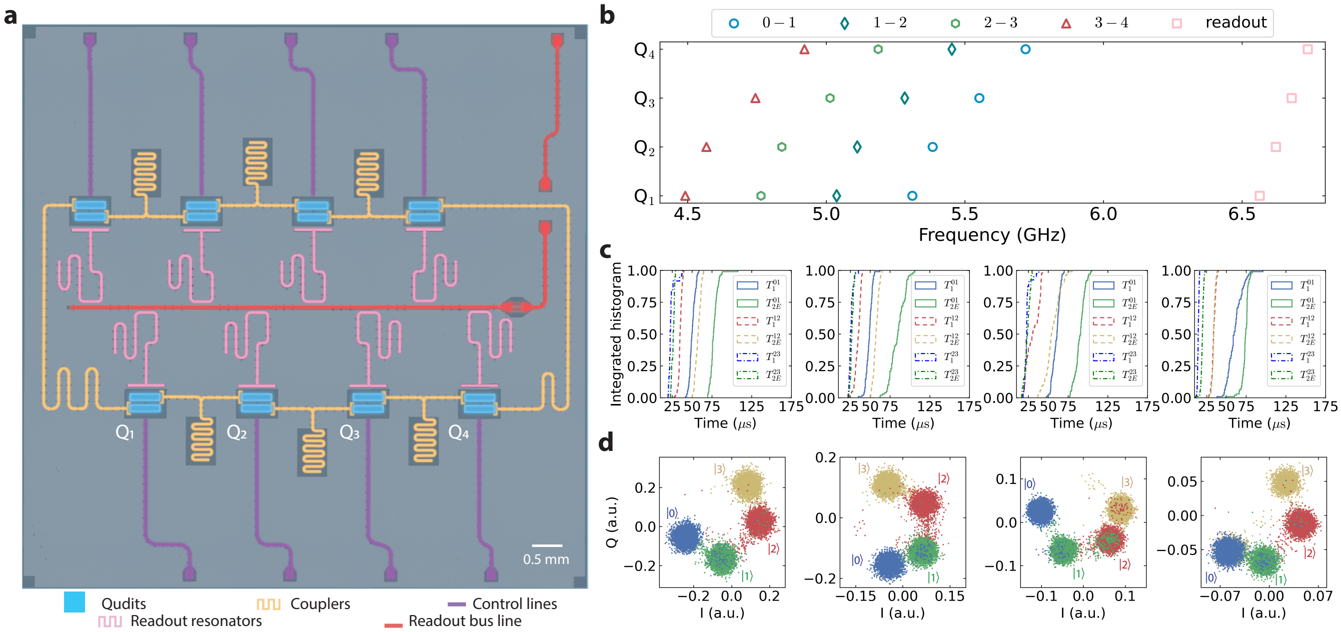

Supplementary Figure 1. Experimental device. (**a**) False-colour micrograph of a representative qudit-ring device. The qudits (blue) are transmon circuits. They are pair-wise connected using resonator couplers (yellow). Each qudit is capacitively connected to a microwave control line (purple) and dispersively coupled to a readout resonator (pink), which are mutually connected to a readout feed line (red). (**b**) Frequency allocation of the qudits used in the experiment. (**c**) Coherence time statistics of the relevant qudit transitions, each one obtained from 100 individual measurements. Data for Q_1 - Q_4 are positioned in order from left to right. (**d**) Dispersive readout histogram of the qudits.

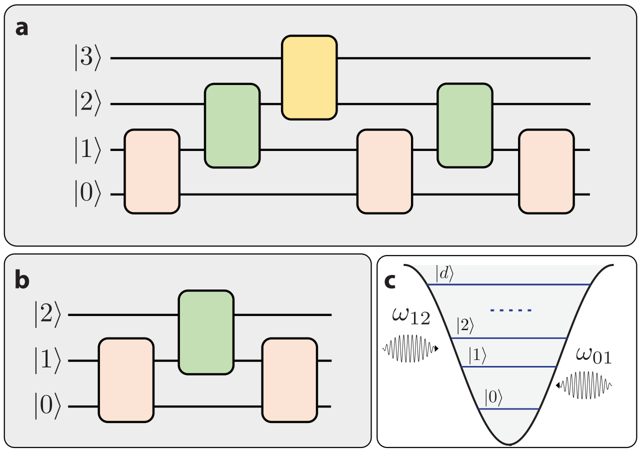

Supplementary Figure 2. Decomposition of control pulses. (**a**) An arbitrary SU(4) qudit gate can be decomposed into 6 blocks of SU(2) unitaries in two-level subspaces of the qudit. (**b**) Similarly, any SU(3) qudit gate can be decomposed using only three subspace SU(2) unitaries. (**c**) Subspace SU(2) operations are performed via resonant microwave drives at frequency $\omega_{k,k+1}$ and virtual-Z gates.

a time-dependent driving term $\epsilon(t)$, we have

$$\hat{H}/\hbar = \omega_{01}\hat{a}^\dagger\hat{a} - \frac{\alpha}{2}\hat{a}^\dagger\hat{a}^\dagger\hat{a}\hat{a} + \epsilon(t)(\hat{a}^\dagger + \hat{a}), \tag{2}$$

where $\hat{a}^\dagger(\hat{a})$ is the bosonic creation (annihilation) operator. In this approximation, the relevant single-photon transition frequencies are $\omega_{k,k+1} = \omega_{01} - k\alpha$. By choosing the driving term $\epsilon(t) = \Omega\cos(\omega_d t)$ with driving frequency ω_d on-resonant with one of the resolvable single photon modes $\omega_{k,k+1}$, we can induce $|k\rangle \leftrightarrow |k+1\rangle$ Rabi oscillations with Rabi rates $\Omega_{k,k+1} = \sqrt{k+1}\Omega$. The characteristics of the enhanced Rabi rate of the higher transitions can be observed in Fig. 1**b** in the main text.

The Rabi interactions naturally generate two-level subspace Pauli-X operations. To realize universal single qudit control and perform Z-like operations, we employ virtual-Z gates which update the phase of the relevant carrier waves at $\omega_{k,k+1}$ [2]. Considering the subspace manifold consisting of d eigenstates in the transmon, we express these native Z operations for a phase update of ϕ as,

$$Z^{k,k+1}(\phi) = \sum_{l=k+1}^{d-1} e^{i\phi}\,|l\rangle\langle l| + \sum_{l=0}^{k} |l\rangle\langle l| \tag{3}$$

where $Z^{k,k+1}$ corresponds to an update to the carrier wave at frequency $\omega_{k,k+1}$.

Compilation and benchmarking of single-qudit gates

To compile arbitrary single-qudit gates over SU(d), we need to tune up only subspace $X90$ pulses corresponding to $\sqrt{X^{k,k+1}}$, where $X^{k,k+1}$ is the two-level embedded qubit Pauli-X operator (or Gell-Mann operator) $X^{k,k+1} = |k\rangle\langle k+1| + |k+1\rangle\langle k|$ for $k \in \mathbb{Z}_{d-1}$. In the case of $d = 2$, any SU(2) gate can be compiled from two $X90$ physical pulses via the ZXZXZ decomposition [2]. As represented in Supplementary Figure 2, from the SU(2) blocks, we can construct SU(d) utilizing 3(6) blocks or 6(12) physical pulses per unitary for $d = 3(4)$ [3]. In Fig. 1b in the main text, randomized benchmarking (RB) [4, 5] was performed over the qudit Clifford group [6] in $d = 2, 3, 4$. The Clifford fidelities were extracted by fitting an exponential decay to the average qudit Z expectation values as a function of the circuit depth. The native gate fidelities were then estimated by dividing the Clifford fidelity by the number of native gates required to implement a qudit Clifford gate in each respective dimension.

Qudit dispersive readout

To measure all qudit states, we couple the transmons dispersively to individual resonator modes. This system can be well approximated by the linear dispersive Hamiltonian given in Ref. [7],

$$\begin{aligned}\hat{H}^D_{JC}/\hbar =&\omega_r \hat{b}^\dagger\hat{b} + \sum_{k=0}^{d} \omega_k |k\rangle\langle k| + \sum_{k=1}^{d-1} \chi_{k-1} |k\rangle\langle k| \\ &- \sum_{k=0}^{d-1} (\chi_k - \chi_{k-1}) |k\rangle\langle k| \hat{b}^\dagger\hat{b},\end{aligned} \tag{4}$$

where $\hat{b}^\dagger(\hat{b})$ is the bosonic creation (annihilation) operator associated with the resonator mode at frequency ω_r. Here, we express the Hamiltonian for d levels of the qudit in its own eigenbasis. Following Supplementary Equation (4), the resonator experiences a dispersive shift of $\chi_k - \chi_{k-1}$ when the qudit is in state $|k\rangle$, where $\chi_k = g_k^2/\Delta_{kr}$. Supplementary Figure 3 shows the phase and amplitude responses of a resonator mode when a dispersively coupled qudit is prepared in $|0\rangle, |1\rangle, |2\rangle$ and $|3\rangle$. For the given χ_k's and resonator linewidths on our device (varying from 1 to 2 MHz), it is possible to choose a probe frequency for each resonator that distinguishes all four relevant qudit states with single shot separability (Supplementary Figure 1**d**).

To extract the readout fidelities in Fig. 1c in the main text, we perform an experiment in which we prepare each qudit cardinal state and then measure and classify the extracted readout signal integrated in the IQ plane according to a Gaussian-mixture-model fitting. This experiment is repeated 2048 times per cardinal state to reduce measurement shot noise. The final averaged results form the so-called confusion matrix comprising of $P(i|j)$'s, where $P(i|j)$ corresponds to the probability of measuring a qudit state $|i\rangle$ after initially preparing a qudit state $|j\rangle$. Finally, the readout fidelities of each qudit state are calculated according to $\mathcal{F}_{|i\rangle} = P(i|i)$, which are the diagonal values of the confusion matrix.

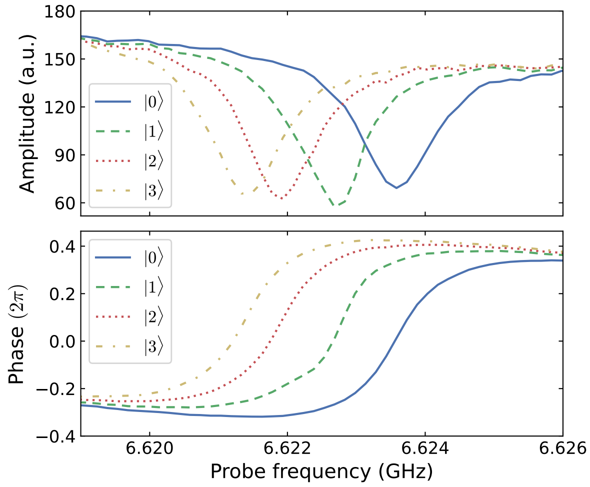

Supplementary Figure 3. **Resonator readout signal**. Magnitude (top) and phase (bottom) of the resonator reflectomery signal when the qudit is prepared in $|0\rangle$, $|1\rangle$, $|2\rangle$, and $|3\rangle$.

Supplementary Note 2 – Theoretical Analysis

We describe here the theoretical framework for the two-photon transitions demonstrated in this work. The two-qudit two-photon transition between states $|k,l\rangle$ and $|k+1,l+1\rangle$ can be understood as a two-qudit generalization of the standard single-atom Raman transition in a "ladder" configuration in which a single-photon transition from the ground state to the second excited state is not possible, but a two-photon transition mediated by a "virtual" energy level is possible. Crucially, this transition avoids populating the first excited state while achieving full population transfer between the ground and second excited states.

Theory of coupled two-level systems

We begin by considering two coupled transmons within the two-level system approximation, i.e. treating them as qubits. We presently derive resonance conditions and transition rates for coherent oscillation between the $|00\rangle$ and $|11\rangle$ states. The static Hamiltonian $\hat{H}_{\rm ss}$ for this system is given as

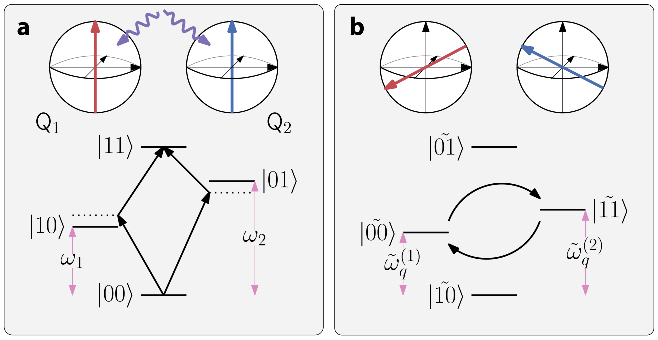

Supplementary Figure 4. Two-photon interaction schematic. (**a**) Level diagram of the two-qubit subspace in the laboratory frame. The two-photon transition between $|00\rangle$ and $|11\rangle$ is enabled by a Raman-like mechanism that avoids transitions outside of the computational subspace. (**b**) Level diagram of the two-qubit subspace in the rotating frame. Although in the laboratory frame, the bare states $|00\rangle$ and $|11\rangle$ appear far from resonance, in the rotating frame, the energies of the corresponding *dressed* states can be tuned into resonance.

$$\begin{aligned}\hat{H}_{\mathrm{ss}} &= \sum_{i=1}^{2} \hat{H}_{\mathrm{q}}^{(i)} + \hat{H}_{\mathrm{c}},\\ \hat{H}_{\mathrm{q}}^{(i)}/\hbar &= -\frac{1}{2}\omega_q^{(i)}\hat{\sigma}_z^{(i)},\\ \hat{H}_{\mathrm{c}}/\hbar &= g\hat{\sigma}_x^{(1)}\hat{\sigma}_x^{(2)},\end{aligned} \tag{5}$$

where $\omega_q^{(i)}$ (used interchangeably with $\omega_{q,i}$) is the transition frequency between the ground state and the first excited state of the i-th qubit and g is the direct qubit-qubit coupling. Without loss of generality, we will work with the assumption $\omega_q^{(1)} < \omega_q^{(2)}$.

We first consider driving both qubits at the same frequency ω_d, generating the Hamiltonian in the laboratory frame as

$$\begin{aligned}\hat{H}_{\mathrm{full}} &= \hat{H}_{\mathrm{ss}} + \sum_{i=1}^{2} \hat{H}_{\mathrm{d}}^{(i)},\\ \hat{H}_{\mathrm{d}}^{(i)}/\hbar &= \Omega_i\left(\hat{\sigma}_+^{(i)} + \hat{\sigma}_-^{(i)}\right)\cos(\omega_d t + \phi_i),\end{aligned} \tag{6}$$

where Ω_i is the amplitude of the drive on qubit i (expressed in terms of the resonant Rabi rate) and ϕ is the relative phase difference between the drives. We also find it convenient to express the results in terms of the "drive ratio" $\lambda \equiv \Omega_1/\Omega_2$, which models crosstalk. The setup in the lab frame is sketched in Supplementary Figure 4**a**, forming a ladder-like Raman configuration in which the direct transition between the $|00\rangle$ and $|11\rangle$ states is forbidden but can be mediated by virtual levels [8, 9].

The formation of the virtual energy levels is better understood in the rotating frame in which the *dressed* energy levels can be tuned to achieve the desired resonance. In the rotating frame, the Hamiltonians read

$$\begin{aligned}\hat{H}_{\mathrm{rot,full}} &= \sum_{i=1}^{2} \hat{H}_{\mathrm{rot,qd}}^{(i)} + \hat{H}_{\mathrm{rot,c}},\\ \hat{H}_{\mathrm{rot,qd}}^{(i)}/\hbar &= -\frac{1}{2}\left(\Delta_i\hat{\sigma}_z^{(i)} + \Omega_i(\hat{\sigma}_+^{(i)}e^{-i\phi_i} + \hat{\sigma}_-^{(i)}e^{i\phi_i})\right),\\ \hat{H}_{\mathrm{rot,c}}/\hbar &= g(\hat{\sigma}_+^{(1)}\hat{\sigma}_-^{(2)} + \hat{\sigma}_-^{(1)}\hat{\sigma}_+^{(2)}),\end{aligned} \tag{7}$$

where the fast-rotating terms have been omitted. Suppose that at $t = 0$, $\Omega_i(t=0) = 0$ and is slowly ramped up to some final value $\Omega_i(t = t_{\mathrm{ramp}}) = \Omega_i$. As in Ref. [10], the eigenstates of the uncoupled, undriven Hamiltonians evolve into those of $\hat{H}_{\mathrm{rot,qd}}^{(i)}$ and are tilted with respect to the z-axis of the Bloch spheres. Defining angles θ_i via

$$\begin{aligned}\cos\theta_i &= \frac{\Delta_i}{\sqrt{\Delta_i^2 + \Omega_i^2}},\\ \sin\theta_i &= \frac{\Omega_i}{\sqrt{\Delta_i^2 + \Omega_i^2}},\end{aligned} \tag{8}$$

the eigenstates of $\hat{H}_{\mathrm{rot,qd}}$ are given by

$$\begin{aligned}|\theta_i\rangle &= \cos\left(\frac{\theta_i}{2}\right)|0\rangle + e^{i\phi_i}\sin\left(\frac{\theta_i}{2}\right)|1\rangle\\ \left|\bar{\theta}_i\right\rangle &= -e^{-i\phi_i}\sin\left(\frac{\theta_i}{2}\right)|0\rangle + \cos\left(\frac{\theta_i}{2}\right)|1\rangle\end{aligned} \tag{9}$$

When $\Delta_i > 0$ ($\Delta_i < 0$) and $\Omega_i(t)$ is ramped up adiabatically from zero, the state $|0\rangle$ evolves into $|\theta\rangle$ ($\left|\bar{\theta}\right\rangle$). The dressed qubit frequency evolves to $\tilde{\omega}_q^{(i)}$, which is given by

$$\tilde{\omega}_q^{(i)} = \mathrm{sgn}(\Delta_i)\sqrt{\Delta_i^2 + \Omega_i^2}. \tag{10}$$

By choosing $\omega_q^{(1)} < \omega_d < \omega_q^{(2)}$, we achieve the level structure shown in Supplementary Figure 4**b**. Then, by adjusting Ω_i, we seek degeneracy between the levels for states $\left|\bar{\theta}\theta\right\rangle$ and $\left|\theta\bar{\theta}\right\rangle$. Solving for this resonance condition, we obtain the following relationship between the resonant drive frequency, the drive amplitude $\Omega \equiv \Omega_2$ to one qudit, the drive ratio $\lambda = \Omega_1/\Omega_2$, and the qudit frequencies,

$$\omega_d = \frac{\omega_q^{(12)}}{2} + \frac{(\lambda^2 - 1)\Omega^2}{2\Delta_q^{(12)}}, \tag{11}$$

where $\omega_q^{(12)} = \omega_q^{(1)} + \omega_q^{(2)}$ and $\Delta_q^{(12)} = \omega_q^{(1)} - \omega_q^{(2)}$. Note that the sign of the shift $\omega_d - \omega_q^{(12)}/2$ is determined by whether the higher or lower frequency qubit is driven more strongly.

Having identified the resonant condition, we enter the frame of $\hat{H}_{\mathrm{rot,qd}}$. The interaction term $\hat{H}_{\mathrm{rot,c}}$ is transformed into

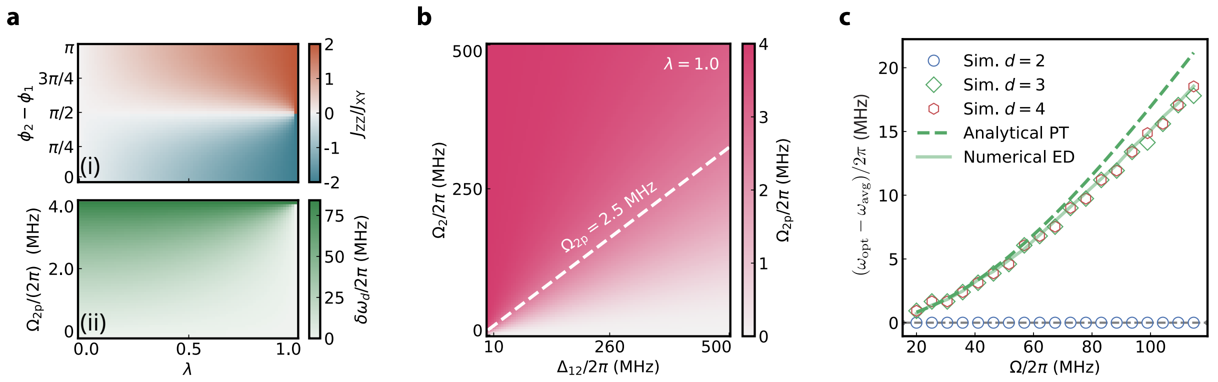

Supplementary Figure 5. **Theoretical results**. (**a**) Tunability of the Hamiltonian through drive ratio and phase. (i) The drive ratio $\lambda = \Omega_1/\Omega_2$ and relative microwave phase $\phi_2 - \phi_1$ of the single qubit drives can be tuned to apply a two-qubit entangling interaction with a variety of relative interaction strengths $J_{\rm ZZ}/J_{\rm XY}$ (ii) The two-photon interaction through monochromatic driving can be activated across the range of frequencies between $\omega_{q,1}$ and $\omega_{q,2}$. For simplicity, we have assumed $\Omega_1 \leq \Omega_2$, so $\omega_d \geq (\omega_{q,1}+\omega_{q,2})/2$, but the opposite detuning can be achieved by driving qudit 1 harder than qudit 2. (**b**) Two-photon swap rate $\Omega_{\rm 2p}$ as a function of the drive amplitude and qudit detuning Δ_{12}, computed in the symmetrically driven regime $\lambda = 1.0$ such that $\omega_d = (\omega_{q,1}+\omega_{q,2})/2$. Even when the energy levels $|k+1,l\rangle$ and $|k,l+1\rangle$ are far-detuned, it is possible to activate the two-photon swap, albeit requiring more drive power. (**c**) Effect of finite anharmonicity on optimal drive frequency. Stark effects from higher levels of the transmon, particularly the third level, shift the dressed qubit frequency and consequently the optimal drive frequency to achieve two-photon transitions. The optimal drive frequency is shown with respect to the average qubit frequency, $\omega_{\rm avg} = (\omega_{q,1}+\omega_{q,2})/2$. Numerical simulations (labeled as "Sim.") were carried out with $d = \{2,3,4\}$ levels for each transmon to identify the optimal drive frequencies and compared to those found by the exact diagonalization (labeled as "Numerical ED") of the rotating frame Hamiltonian and analytical perturbation theory (labeled as "Analytical PT").

$$\begin{aligned}
\tilde{H}_c/\hbar &= J_I\left(\tilde{\sigma}_x^{(1)}\tilde{\sigma}_x^{(2)} - \tilde{\sigma}_y^{(1)}\tilde{\sigma}_y^{(2)}\right) + J_Q\left(\tilde{\sigma}_x^{(1)}\tilde{\sigma}_y^{(2)} + \tilde{\sigma}_y^{(1)}\tilde{\sigma}_x^{(2)}\right) + J_{\rm ZZ}\tilde{\sigma}_z^{(1)}\tilde{\sigma}_z^{(2)},\\
J_I &= \frac{g}{8}\Big[(1+\cos\theta_1)(1+\cos\theta_2)\cos 2\phi_1 + (1-\cos\theta_1)(1-\cos\theta_2)\cos 2\phi_2\Big],\\
J_Q &= -\frac{g}{8}\Big[(1+\cos\theta_1)(1+\cos\theta_2)\sin 2\phi_1 + (1-\cos\theta_1)(1-\cos\theta_2)\sin 2\phi_2\Big],\\
J_{\rm ZZ} &= -\frac{g}{2}\sin(\theta_1)\sin(\theta_2)\cos(\phi_1-\phi_2).
\end{aligned} \tag{12}$$

As expected, the maximal oscillation rate $\Omega_{\rm 2p}$ in the $00-11$ subspace, $4\sqrt{J_I^2+J_Q^2}$, is set by the bare coupling g. This asymptotic behavior is well-captured by this theoretical approach, which, in contrast to prior work, treats the coupling term $\hat{H}_{\rm c}$ *after* exactly diagonalizing the full uncoupled rotating Hamiltonian $\hat{H}_{\rm rot,qd}$ rather than diagonalizing the full, coupled static Hamiltonian first and then treating the driving terms perturbatively, such as in Ref. [11]. In that work, the swap rate $\Omega_{\rm 2p}$ acquires significant corrections due to the close frequency spacing of the $|11\rangle$ and $|02\rangle$ states, and only the limit of low drive power is considered. However, standard expressions for $\Omega_{\rm 2p}$ in the low-power limit, including the pure qubit limit of Ref. [11], can be recovered by Taylor expansion of Supplementary Equations (12). This alternative approach is justified due to the unique hierarchy of energy scales that arises from entangling qubits via strong off-resonant drives: the parameter Ω_i/Δ_i is no longer "small", so corrections to the perturbation theory are significant.

To build intuition for the expressions in Supplementary Equations (11) and (12), we consider two limiting cases. First, when the qubits are driven symmetrically ($\lambda = 1$) and in-phase with the same amplitude Ω, the rates are

simplified to

$$\begin{aligned}\omega_d &= \frac{\omega_q^{(12)}}{2}\\ J_I &= \frac{g}{4}\frac{\Omega^2}{\Omega^2+(\Delta_q^{(12)})^2/4},\\ J_Q &= 0,\\ J_{\mathrm{ZZ}} &= -\frac{g}{2}\frac{\Omega^2}{\Omega^2+(\Delta_q^{(12)})^2/4}.\end{aligned} \tag{13}$$

On the other hand, when only one qubit is driven (e.g. $\lambda = 0$) as done in Ref. [12], we obtain

$$\begin{aligned}\omega_d &= \frac{\omega_q^{(12)}}{2} - \frac{\Omega^2}{2\Delta_q^{(12)}},\\ J_I &= \frac{g}{4}\left(1-\frac{\Delta_q^{(12)}/2-\Omega^2/\Delta_q^{(12)}}{\sqrt{\Omega^2+(\Delta_q^{(12)}/2-\Omega^2/\Delta_q^{(12)})^2}}\right)\\ J_Q &= 0,\\ J_{\mathrm{ZZ}} &= 0.\end{aligned} \tag{14}$$

Supplementary Equations (11) and (12) suggest that by controlling the drive ratio λ and the relative phase of the microwave drives on each transmon, $\phi = \phi_2 - \phi_1$, one can control (i) the ratio of the two-photon excitation term $J_{\mathrm{XY}} = \sqrt{J_I^2 + J_Q^2}$ to the ZZ term J_{ZZ}, as well as (ii) the optimal drive frequency ω_d. We demonstrate the flexibility of this control in Supplementary Figure 5**a**. We speculate that this control could be useful for performing quantum simulations of many-body systems with nearest-neighbor couplings using fewer two-qubit gates, as one can tune ratios of the interaction terms in the Hamiltonian easily.

Our analysis has thus far concentrated on the two-photon transition between the $|00\rangle$ and $|11\rangle$ states of a system of two coupled qubits. However, we can apply a similar analysis to subspaces consisting of higher levels to obtain rates for general high-dimensional systems, assuming that all other levels are far enough apart that they do not contribute significantly to the dynamics. To obtain the expressions for the $|k,l\rangle \to |k+1,l+1\rangle$ transition, the detunings Δ_i and $\Delta_q^{(12)}$ are now defined relative to the $k \to (k+1)$ and $l \to (l+1)$ transition frequencies of the qudit pair. The drive amplitude Ω_1 (Ω_2) is defined with respect to the Rabi rate in the $|k\rangle, |k+1\rangle$ ($|l\rangle, |l+1\rangle$) subspace when this transition is driven resonantly, and g is replaced by the bare coupling between states $|k,l+1\rangle$ and $|k+1,l\rangle$. The latter typically scales as $\sqrt{(k+1)(l+1)}$ due to the matrix elements of the bosonic ladder operator. We therefore fit the experimental data for the measured resonance frequencies ω_d and swap rates $\Omega_{2\mathrm{p}}$ in Fig. 2**c-d** using Supplementary Equations (11) and (12) as ansätze with an additional parameter to convert voltage output on drive lines to Rabi rates $\{\Omega_1, \Omega_2\}$. The extracted drive amplitude ratio is $\lambda = \{0.64, 0.63, 0.66\}$ for the $\{00-11, 11-22, 22-33\}$ transitions.

Finally, we note that even when the single-photon transition energies from $|k,l\rangle$ to $|k+1,l\rangle$ and $|k,l+1\rangle$ are far-detuned, it is still possible to activate the two-photon transition from $|k,l\rangle$ to $|k+1,l+1\rangle$, although a larger drive amplitude (in the relevant qudit subspaces) is probably needed. We illustrate this point in Supplementary Figure 5**b**, which shows the two-photon swap rate $\Omega_{2\mathrm{p}}$ as a function of the drive amplitude Ω_2 and the difference in single photon transition energies Δ_{12} in the symmetrically driven regime.

Finite anharmonic correction

The preceding analysis of the $|00\rangle \to |11\rangle$ two-photon transition assumes that states outside the computational subspace are sufficiently off-resonant such that they do not contribute significantly to the dynamics, which is a good assumption for systems with high anharmonicity [9, 13]. However, deviations from this theory manifest when higher levels of the transmon are taken into account. The strong off-resonant drive induces a Stark shift of the dressed qubit frequency in Supplementary Equation (10), which must be taken into account when solving for the optimal drive frequency ω_d at a given drive amplitude Ω.

We examine this effect by performing numerical simulation of two symmetrically driven transmons using QuTiP [14], retaining up to four energy levels per transmon as shown in Supplementary Figure 5**c**. As predicted by Supplementary Equation (11), when only two levels are retained, the optimal frequency ω_d is independent of the drive strength Ω. However, when higher levels are taken into account, a nontrivial dependence manifests. This dependence can be understood by considering the Hamiltonian of the three-level Duffing Oscillator in the rotating frame of the drive for each transmon separately,

$$\hat{H}_i/\hbar = \left(\Delta_i - \frac{\alpha_i}{2}\right)\hat{a}_i^\dagger\hat{a}_i + \frac{\alpha_i}{2}(\hat{a}_i^\dagger\hat{a}_i)^2 + \frac{\Omega_i}{2}\left(\hat{a}_i + \hat{a}_i^\dagger\right), \tag{15}$$

where α_i is the anharmonicity of qudit i, a_i is the bosonic annihilation operator for the modes of qudit i, and the fast-rotating terms have been dropped. By considering Ω_i as a parameter which is slowly turned on from 0 at $t = 0$ to their final values Ω_i at $t = t_{\mathrm{ramp}}$, one can follow the eigenstates of the rotating frame Hamiltonian from $t = 0$ to $t = t_{\mathrm{ramp}}$ and compute the energy difference $\tilde{\omega}_q^{(i)}$ between the states $|0\rangle$ and $|1\rangle$. This generalizes the dressed qubit frequency introduced in Supplementary Equation (10). Similarly, the resonance between the $|00\rangle$ and $|11\rangle$ states is achieved at a given drive power when ω_d is tuned such that $\tilde{\omega}_q^{(1)} + \tilde{\omega}_q^{(2)} = 0$. The entire procedure

of diagonalization and solution for ω_d can be carried out through exact diagonalization of Supplementary Equation (15) using linear algebra software (Numerical ED) or by standard Schrieffer-Wolff perturbation theory (Analytical PT) to obtain expressions for $\{\tilde{\omega}_q^{(i)}\}$ and then again using numerics to solve for resonance. We compare these two approaches to the optimal frequency obtained by simulations of spectroscopy in QuTiP [14] in Supplementary Figure 5**c**. The agreement between the numerical simulation and the semi-analytical approaches for $d > 2$ suggests that the nontrivial dependence of ω_d on Ω can indeed be understood through the framework of effective transition frequencies used in this work.

Adiabaticity requirements

The adiabaticity condition can be understood as arising from the need to transform from the laboratory frame to the dressed frame. Specifically, consider the Hamiltonian of a single, off-resonantly driven qubit in the rotating frame of the drive: $H(t) = \Delta_{\rm q}\sigma^z + \Omega_R(t)\sigma^x$. The drive has an envelope $\Omega_R(t)$. Due to the quantum adiabatic theorem, if Ω_R is slowly ramped up or down, the eigenstates of σ^z will evolve into the instantaneous eigenstates of $H(t)$. In practice, it is important to change $H(t)$ sufficiently slowly. Failure to do so leads to coherent errors which can be understood as Landau-Zener transitions. The relevant parameter in such a transition is the rate at which the spectral gap of H is changing, which is, to low order in $\Omega_R/\Delta_{\rm q}$, $\sim \Omega_R\dot{\Omega}_R/\Delta_{\rm q}$. This tells us that at fixed $\Delta_{\rm q}$ and Ω_R, we must decrease $\dot{\Omega}_R$ to fulfill adiabaticity as much as possible, e.g. by slowly ramping up and down the drives. However, as with other gate schemes, a slower gate generally results in greater incoherent error, so the ramps should be chosen to balance these two types of error. Notably, Ref. [10] showed that shortcut-to-adiabaticity techniques can be used to effectively reduce the ramping time. Future works exploring the diabatic regime are also of immense interest, and such advances can be applied to operate fast gates using two-photon interactions in fixed-frequency superconducting circuits.

Supplementary Note 3 – Extended Two-photon Interaction Data

The two-photon interaction can also be realized between $|k,l\rangle$ and $|k+1,l+1\rangle$ for $k \neq l$. To provide the supporting data, we showcase the microwave-driven exchange interaction between $|01\rangle$ and $|12\rangle$. The first evidence appears in a spectroscopy measurement in which we prepare the qudits to be in $|01\rangle$ and then sweep the drive frequency across a wide range, as shown in Supplementary Figure 6**a**. Using the single-shot readout capability, we can deterministically compute the probability of the qudits in specific states after applying the drives at each frequency point.

The coherent population transfer is then further verified by performing a time-domain measurement of the qudit population after applying pulses with varying durations. As the drive frequency and duration are swept across the preliminary data point in the spectral domain, the exchange interaction manifests as a chevron pattern in Supplementary Figure 6**b**. Fixing the drive frequency at the optimal (symmetric) point, we can inspect the microwave-induced oscillation between $|01\rangle$ and $|12\rangle$, as shown in Supplementary Figure 6**c**.

Supplementary Note 4 – Extended Data for CnZ gates

We leverage Cross-Entropy Benchmarking (XEB) [15] to further characterize the multi-qubit operation. The cross-entropy $H(p_1, p_2)$ is a statistical measure of the similarity between two probability distributions p_1 and p_2. In characterizing the fidelity of a quantum operation, we consider the probability distribution $p(x)$ giving the probability of measuring output bitstring x after applying the operation.

Let us define p as the ideal probability, q as the experimental one, then the XEB circuit fidelity is given as

$$\mathcal{F}_{\rm XEB} = \frac{H(p,q) - H(p,u)}{H(p,p) - H(p,u)} \equiv \frac{m_U - u_U}{e_U - u_U}, \quad (16)$$

where $H(p_1, p_2) = \sum_x p(x)p_2(x)$ is the linear cross-entropy between two probability distributions $p_1(x)$ and $p_2(x)$, and $u(x) = 1/d$ is the uniform probability distribution on the bitstrings. The concept of fidelity in this context can be interpreted as the difference in the ideal to measured and ideal to uniform cross entropies, normalized by the difference if the measured distribution was to perfectly match the ideal distribution.

For a randomized circuit, the error can be viewed as the deviation of the measured bitstring distributions from a uniform distribution. Notably, XEB does not involve finding the inverse gate. Therefore, it is a versatile tool to characterize non-Clifford and multi-qubit gates such as those in the CnZ gate family. By interleaving the implemented gate with SU(2) local rotations, we can tailor the gate errors into a global depolarizing channel.

Performing XEB measurement, we obtain the dressed cycle fidelity at different cycle depths. Fitting this to the simple depolarizing noise model gives us the average fidelity at every depth. By varying the depth, we can extract the average cycle fidelity, as shown in Supplementary Figure 7. The dressed cycle fidelity for the CCZ gate is 91.9(3)%, approximately equal to the dressed cycle fidelity given by the CB method (92.44(6)%), albeit with higher uncertainty. This implies that we

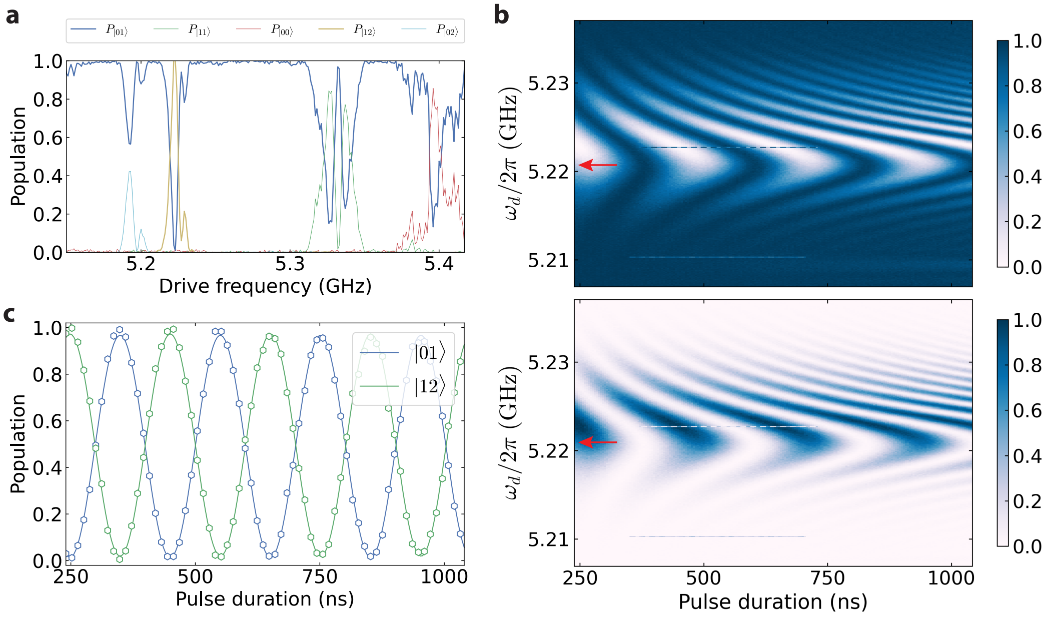

Supplementary Figure 6. $|01\rangle \leftrightarrow |12\rangle$ **interaction**. (**a**) Transition spectrum of a pair of qudits prepared in $|01\rangle$, revealing the sign of the transition. (**b**) Chevron pattern of the transition with varying drive frequency and pulse duration. (**c**) Coherent driven oscillation between $|01\rangle$ and $|12\rangle$ originating from the two-photon dynamics. The drive frequency is indicated by the red arrows in panel **b**.

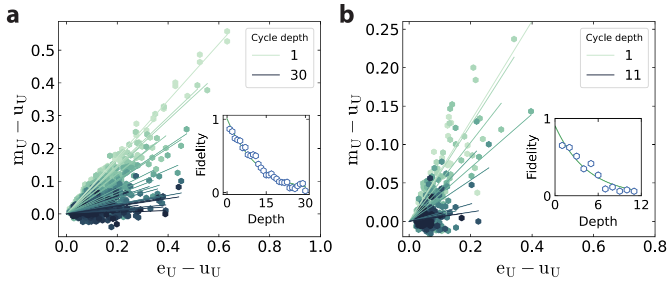

Supplementary Figure 7. **Cross-entropy benchmarking**. (**a**) CCZ result. The differences in the normalized linear cross-entropies for ideal ($m_U - u_U$) and measured ($e_U - u_U$) are shown, which can be used to extract the cycle fidelity. Varying the cycle depth results in an exponential decay (inset) that embodies the dressed cycle fidelity. (**b**) CCCZ result. The increased cycle error cuts down the maximum cycle depth required.

can roughly estimate the gate fidelity by combining the XEB fidelity with the CB reference cycle fidelity. This approach allows us to estimate the CCCZ gate fidelity to be 88(2)%, lower than the truth table fidelity. This implies a finite correlated phase error in the obtained result. We note that our numerical simulation shows that the small phase shift of the target qubit associated with the control state $|111\rangle_c$ accounts for an error of less than 0.5%.

Supplementary Note 5 – Quantum Tomography of High-dimensional States

To characterize the fidelity of the realized quantum states, we utilize quantum state tomography which has been previously applied to isolated [7, 16, 17] as well as multipartite qudit states [18–20]. To reconstruct an arbitrary single qudit state, we perform projections over an informationally complete set of local rotations. To limit the effects of the state preparation and measurement (SPAM) errors, we choose these to be closest to the native gate set of our device. We note that the Gell-Mann operators, given by $X^{k,l} = |k\rangle\langle l| + |l\rangle\langle k|$ and $Y^{k,l} = i(|k\rangle\langle l| - |l\rangle\langle k|)$, correspond to the native rotations induced by Rabi oscillations at $\omega_{k,k+1}$ for the case of $l = k+1$. We thus choose the natural set of local projections for state tomography as projections onto all the computational states $|0\rangle, \ldots |d-1\rangle$ as well as two-level $\sqrt{X^{k,k+1}}$ and $\sqrt{Y^{k,k+1}}$ projections within each subspace, or explicitly, the set of projectors M given by

$$\begin{aligned} M =\{ & I, X^{0,1}, X^{1,2}X^{0,1}, X^{2,3}X^{1,2}X^{0,1}, \sqrt{X^{0,1}}, \sqrt{Y^{0,1}}, \\ & \sqrt{X^{1,2}}X^{0,1}, \sqrt{Y^{1,2}}X^{0,1}, X^{1,2}\sqrt{X^{0,1}}, X^{1,2}\sqrt{Y^{0,1}}, \\ & X^{2,3}X^{1,2}\sqrt{X^{0,1}}, X^{2,3}X^{1,2}\sqrt{Y^{0,1}}, X^{2,3}\sqrt{X^{1,2}}X^{0,1}, \\ & X^{2,3}\sqrt{Y^{1,2}}X^{0,1}, \sqrt{X^{2,3}}X^{1,2}X^{0,1}, \sqrt{Y^{2,3}}X^{1,2}X^{0,1}\}. \end{aligned} \tag{17}$$

For the projections of an n-qudit state, we simply apply the projections in $M_n = M^{\otimes n}$.

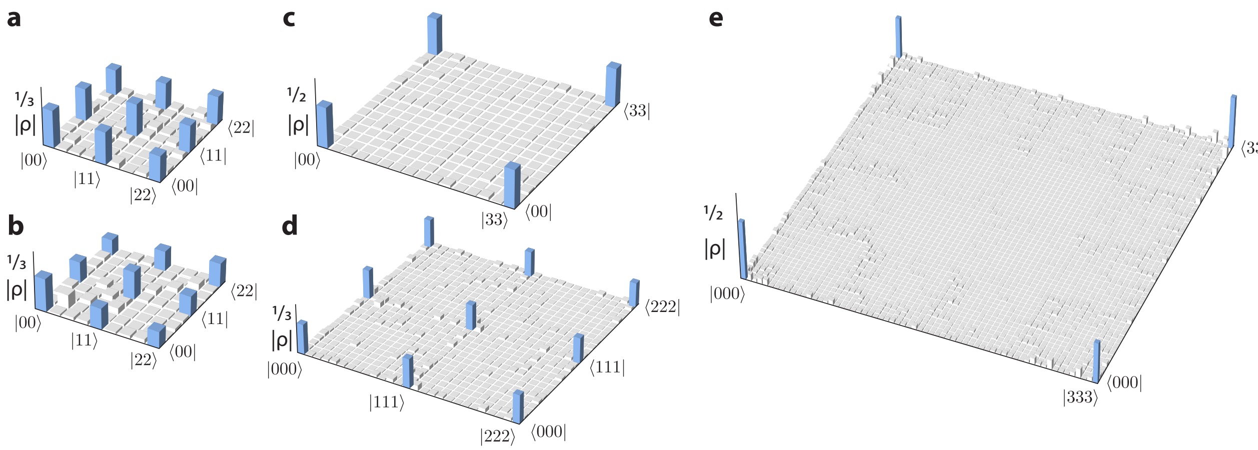

Supplementary Figure 8. **Extended tomography results**. (**a**) Q_1-Q_3 qutrit Bell state. The raw (purified) fidelity is 75.7% (96.3%). (**b**) Q_1-Q_4 qutrit Bell state with $\mathcal{F} = 53.0\%$ (95.7%). (**c**) Schrödinger cat state $(|00\rangle + |33\rangle)/\sqrt{2}$ with $\mathcal{F} = 98.6\%$ (99.3%). (**d**) High-dimensional GHZ state with $n = d = 3$ and $\mathcal{F} = 90.8\%$ (96.4%). (**e**) Schrödinger cat state $(|000\rangle + |333\rangle)/\sqrt{2}$ with $\mathcal{F} = 80.1\%$ (90.9%).

To further mitigate the effects of SPAM measurements, we characterize the readout confusion matrix (or readout misassignment matrix see Fig. 1**b**) over the relevant n qudit states $\{|0\rangle \dots |d-1\rangle\}^{\otimes n}$. Given the fidelity of our native operations characterized by RB, we can assume that the readout assignment errors are dominant. This allows us to account for the measurement errors by applying the inverse of the confusion matrix to our averaged ditstring results. Then, the qudit density matrices are reconstructed via maximum likelihood estimation (MLE). In addition to the results presented in Figures 4 and 5 in the main text, the reconstructed density matrices of the non-local qutrit Bell states between Q_1-Q_3 and Q_1-Q_4, two and three qudit cat states, and three qutrit GHZ states can all be found in Supplementary Figure 8. The state fidelity is computed following the relation

$$\mathcal{F}(\rho, \sigma) = \mathrm{Tr}\left(\sqrt{\rho}\sigma\sqrt{\rho}\right) \tag{18}$$

where ρ is the experimentally reconstructed density matrix, and σ is the ideal density matrix. Finally, we note that the diagonal terms in the density matrix can be measured directly to reduce fluctuation effects arising during the long tomography measurement.

Supplementary Note 6 – Errors and Uncertainties in Quantum State Tomography

In this study, the assessment of entangled multidimensional states is conducted through quantum state tomography (QST), a method that reconstructs the density matrices of states using measurements of Pauli operators and MLE. To evaluate the effectiveness of this approach, it is crucial to comprehend the inherent noise in QST and its dependence on the dimensions of the qudits, denoted as d, and the number of qudits, denoted as n. Specifically, we delve into the impact of shot noise, an inherent element of the QST procedure. This noise arises from the discrepancy between the sample expectation value and the true expectation value, a consequence of a finite number of measurement repetitions (or shots) denoted as n_{rep}. As per the Central Limit Theorem, the shot noise scales with $1/\sqrt{n_{\mathrm{rep}}}$ when $n_{\mathrm{rep}} \gg 1$.

First, we investigate the scaling behavior of shot noise with an increasing qudit dimension d. This is accomplished by simulating tomography experiments for qudit Bell states $\mathrm{Bell}_2, \mathrm{Bell}_3, \mathrm{Bell4}$ with $d = \{2, 3, 4\}$. The accumulative probability distribution of the fidelities between the QST-reconstructed density matrix and the corresponding Bell state is shown in Supplementary Figure 9**a**. The average fidelities are 0.987, 0.976, 0.963, with the standard deviations of 0.004, 0.006, 0.008, respectively, where each distribution consists of 100 simulations of a tomography experiment with $n_{\mathrm{rep}} = 1000$. The bias of the average fidelities away from 1 is attributed to the MLE process [21], while the standard deviation arises from shot noise. Despite the exponential enlargement of the Hilbert space with the increasing qudit dimension d, the shot noise is expected to increase only quadratically with d due to the entangling structure of the Bell states [22].

Then, we note that increasing the number of qudits also increases the Hilbert space dimension. With a similar method, we estimate the shot noise of the QST for GHZ states involving 2, 3 and 4 qubits ($d = 2$) and show their accumulative probability distribution in Supplementary Figure 9**b**. The average fidelities are 0.987, 0.976, 0.961, and the standard deviations are 0.004, 0.003, 0.006. We observe no significant increase or clear

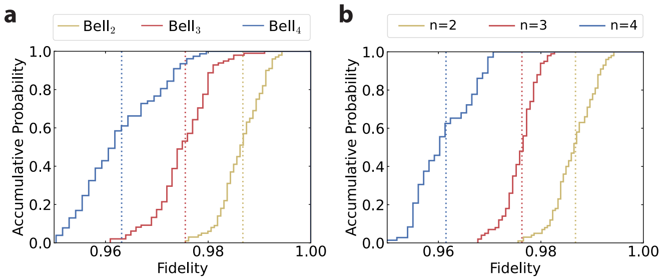

Supplementary Figure 9. Shot noise simulation. (**a**) Accumulative probability distribution for qudit Bell states $\text{Bell}_2, \text{Bell}_3, \text{Bell}_4$ with $d = 2, 3, 4$, where the fidelities are estimated to be 0.987(4), 0.976(6), 0.963(8). (**b**) Accumulative probability distribution for 2 qubit, 3 qubit and 4 qubit GHZ states, where the fidelities are estimated to be 0.987(4), 0.976(3), 0.96(6).

scaling relations between the shot noise and the qubit numbers, which can be explained by the entangling structure of the GHZ states [22].

This stems from the fact that the fidelity of the Bell states is predominantly determined by the population terms $\langle i \dots i|\rho|i \dots i\rangle$ and the coherence terms $\langle i \dots i|\rho|j \dots j\rangle$, so the uncertainties remain the same for larger systems [22]. Meanwhile, the number of important terms in the fidelity increases with the number of constituents in the state, which explains the previous observation. We note that our experiment involves entangled states with up to 4 constituents, and thus, the simulation of shot noise for Bell_d with $d = 2, 3, 4$ encompasses the important standard deviations for all the reported results.

To visualize the constructed quantum states, we use the bar plots as shown through out the text. To emphasize the important terms of the density matrices that contribute to the state fidelity, we apply a threshold to the bars in each plot. If the values are below this threshold, the bars are plotted in grey. Otherwise, they are plotted in blue. If the fidelities of the constructed states are sufficiently high, all the important terms are highlighted against the pastel background. For the figures presented, we choose each threshold to be half of the highest ideal value of the corresponding matrix. This allows the important entries to be visualized effectively.

State preparation and measurement (SPAM) errors play an important role in the context of state tomography experiments. In our work, the effective state preparation step is performed by waiting long enough between each circuit (300 μs) for the qudits to decay to a thermal equilibrium state (nearly equal to the $|0\rangle$ state) and then performing a simultaneous heralding measurement to ensure all qudits are in the ground state. This step is performed with high-fidelity owing to the above 99% $|0\rangle$ state readout fidelity of each qudit. The other sources of potential SPAM errors correspond to projection errors in the local rotation gates and readout assignment errors. We effectively mitigate readout assignment errors by inverting the qudit readout confusion matrices when reconstructing the density matrices [10, 18].

Notably, the other major source of errors in terms of state fidelity can therefore correspond to decoherence, projection errors, and errors in the two-photon interactions employed to generate the entangled-qudit states. We effectively cancel the decoherence errors by numerically purifying our density matrix [23]. The remaining state fidelity errors can be attributed to errors in our two-photon gates and from errors induced by crosstalk in performing our local tomographic pulses. As the problem of both classical (stray microwave field) and quantum crosstalk (cross-Kerr interaction [19]) grows with dimension d, we expect more deleterious effects of imperfect tomographic pulses for higher dimensional qudit states in the present work. In the future, we seek to mitigate these nonidealities by implementing a tunable coupling architecture [24], applying dynamical decoupling techniques [25] to qudits, and leveraging 3D integrated fabrication [26] to effectively suppress the quantum and classical crosstalks.

Supplementary Note 7 – Phase-space Description of High-dimensional States

In the realm of quantum mechanics, the choice of a representation is often guided by practical considerations. In this vein, the concept of coherent states has emerged as a fundamental framework for standard descriptions within the field of quantum optics, owing to their attractive characteristics: (i) They are evolved from the vacuum state by a unitary operator, and are minimum-uncertainty states. (ii) They obey a completeness relation and thus form a good set of basis states. (iii) They provide a quantum approach to conceptualize classical fields. Notably, macroscopic quantum states of atomic ensembles can also be described by such a concept. This duality is related to the group contraction of spin operators based on the angular momentum algebra to bosonic operators based on the harmonic-oscillator algebra [27].

A *spin coherent state* (SCS) $|\theta, \phi\rangle$ is defined as an eigenstate of a spin component in the (θ, ϕ) direction, $S_{\theta,\phi} = S_x \sin\theta\cos\phi + S_y \sin\theta\sin\phi + S_z\cos\theta$. Here, θ denotes the polar angle, ϕ denotes the azimuthal angle, and the spin system obeys the cyclic commutation relation $[S_i, S_j] = i\epsilon_{ijk}S_k$, where ϵ_k is the Levi-Civita symbol. The associated uncertainty relation is $\langle\Delta S_i^2\rangle\langle\Delta S_j^2\rangle \geq \frac{1}{4}|\langle S_k\rangle|^2$. An SCS satisfies the minimal uncertainty relation with equal uncertainties in both orthogonal components, corresponding to an isotropic Husimi-Q quasiprobability distribution in the spherical phase space [27, 28], as shown in Supplementary Figure 10**a**. In the present framework, we project a high-

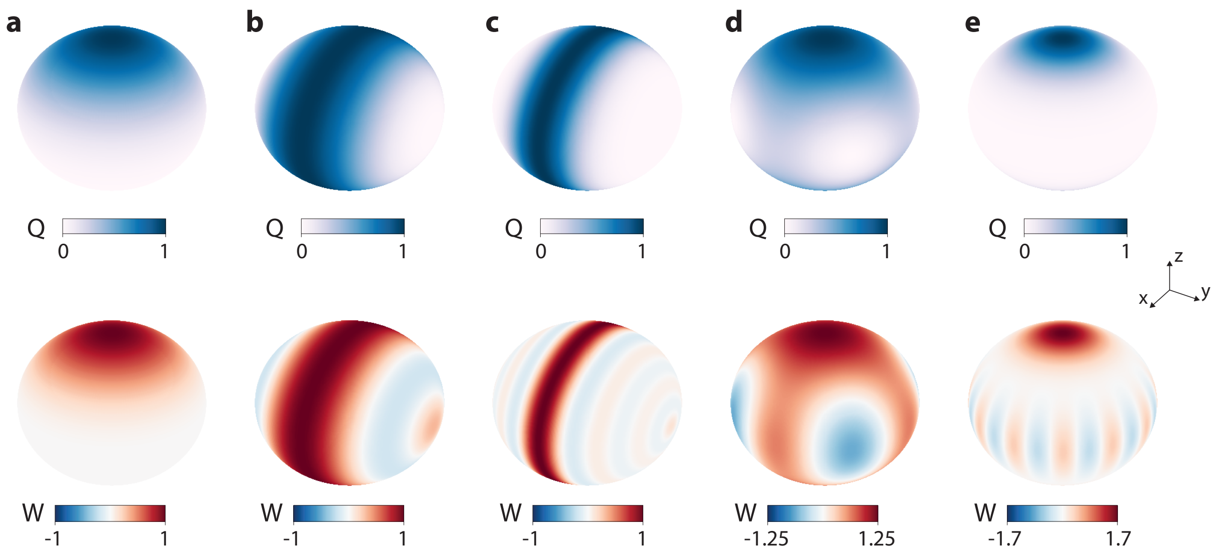

Supplementary Figure 10. **Quasiprobability distributions of high-dimensional states**. The top row shows the Husimi-Q distributions. The bottom row shows the Wigner functions. (**a**) Coherent state $|00\rangle$ with $d=3$. (**b**) Bell_3 state. (**c**) Bell_7 state. (**d**) Cat_3 state with $n=2$. (**e**) Cat_7 state with $n=2$.

dimensional spin-j state $|j\rangle$, where $j=\frac{1}{2}(d-1)$, onto an SCS for useful visualization using the generalized Bloch sphere,

$$|\theta,\phi\rangle=\exp\left[\frac{1}{2}\theta(e^{i\phi}J_{-}-e^{-i\phi}J_{+})\right]|j\rangle, \tag{19}$$

where $J_{\pm}=J_x\pm iJ_y$ are the raising/lowering angular momentum operators, and we denote $|j\rangle\equiv|j,m=j\rangle$ for a general spin-j system [27].

An ensemble of n pure SCS's simply form a product state,

$$\prod_{k=0}^{n-1}|\theta_k,\phi_k\rangle_k=\prod_{k=0}^{n-1}\left[\cos\frac{\theta_k}{2}|0\rangle_k+\sin\frac{\theta_k}{2}e^{i\phi_k}|1\rangle_k\right], \tag{20}$$

where $|0\rangle_k$ and $|1\rangle_k$ are eigenstates of S_z in the kth SCS. If all the individual constituents are aligned along the same direction (θ,ϕ), the resulting state becomes the collective SCS,

$$|\theta,\phi\rangle=\left[\cos\frac{\theta}{2}|0\rangle+\sin\frac{\theta}{2}e^{i\phi}|1\rangle\right]^{\otimes n}. \tag{21}$$

This alignment can be conveniently realized via initialization of all the qudits to the same state. For a collection of spin-j system with dimension $d+1$, if the basis SCS is projected from $|jj\ldots j\rangle$ with $j=d/2$, then the high-dimensional states with isotropic QPD are $|00\ldots0\rangle$ and $|dd\ldots d\rangle$.

Starting from the optical coherent state, various interesting states of light can be realized. We presently explore the duality of these states in qudit systems starting from the SCS. An important concept in quantum optics is *squeezing*, which is paradigmatically generated by the operator $\hat{\mathcal{S}}(\zeta)=\exp\left[\frac{1}{2}(\zeta\hat{a}^{\dagger2}-\zeta^{*}\hat{a}^{2})\right]$, where ζ is the squeezing parameter and $\hat{a}$ ($\hat{a}^{\dagger}$) is the bosonic annihilation (creation) operator. Specifically, the photonic two-mode squeezing process reads $\hat{\mathcal{S}}(\zeta)=\exp(\zeta\hat{a}^{\dagger}\hat{b}^{\dagger}-\zeta^{*}\hat{a}\hat{b})$, where ζ is the squeezing strength, and $\hat{a}$ ($\hat{b}$) is the annihilation operator of the first (second) mode.

The process results in the two-mode squeezed state, $|\Psi\rangle_{\text{2ms}}\propto\sum_{N=0}^{\infty}c_N|NN\rangle$, where c_N is a coefficient dependent on ζ and N is the quanta number [29]. In a coupled system comprising of two qudits with dimension d, if we draw the duality of N ranging from 0 to $d-1$, the high-dimensional Bell_d state would be close to spin squeezed state (SSS). As opposed to the SCS, the SSS is signified by the shrinking of its Husimi-Q distribution along a geodesic, which is further enhanced by increasing the dimension of the system, as shown in Supplementary Figure 10**b**,**c**.

Another important bosonic state of immense interest is the Schrödinger cat state. In atomic systems, this represents a superposition of macroscopic SCS's. The structure of high-dimensional systems and the two-photon dynamics suggest that Schrödinger cat states can be realized efficiently in qudits. We demonstrate this by creating the qudit Schrödinger cat states, which are superpositions of the qudit coherent states, as discussed in the main text. Importantly, the size of the realized cat depends equally on the dimension d and the number of parties n. We provide a gallery of Wigner functions for different cat states in Supplementary Figure 10**d**,**e**.

Quasiprobability distributions

The concepts of squeezing and macroscopic superpositions are more conveniently represented using quasiprobability distribution functions in phase space, such as the Husimi-Q function or the Wigner function. In particular, the Husimi-Q distribution is frequently employed to display the macroscopic aspect of entangled quantum systems, due to its relatively straightforward calculation procedure via the formula

$$Q(\theta,\phi) \propto \langle\theta,\phi|\hat{\rho}|\theta,\phi\rangle. \tag{22}$$

This involves computing the overlap probability between the target state specified by $\hat{\rho}$ and the SCS $|\theta,\phi\rangle$ as the basis state. The Husimi-Q function is nevertheless not suitable to exhibit the non-classical feature of macroscopic superposition states such as the Schrödinger cats, as showcased in Supplementary Figure 10**d,e**.

The Wigner function's ability to take on negative values, on the other hand, has proven to be transformative in the visualization of quantum correlations. It has thereby become an indispensible tool in the analysis of cat states. The Wigner function can be viewed as the expectation value of a normalized parity operator, which renders it possible to be represented using different bases. Here, we follow the convention using tensor product kernels prescribed by Ref. [30],

$$W_{\hat{\rho}}(\theta,\phi) = \langle\hat{U}(\theta,\phi)\hat{\Pi}\hat{U}^{\dagger}(\theta,\phi)\rangle_{\hat{\rho}}. \tag{23}$$

Here, $\hat{\rho}$ is the density matrix, $\hat{U}$ is the general displacement or rotation operator, and $\hat{\Pi}$ can be viewed as the spin parity operator, analogous to the bosonic case. These operators are not unique, and they only have to obey the Stratonovich-Weyl correspondence. Our capability to measure high-dimensional systems motivates the use of a tensor product of spins. This simply involves laying out the SU(d) rotation of a single-qudit, using $\hat{\Pi}_{\otimes^n \mathrm{SU}(d)} = \otimes^n \hat{\Pi}_{\mathrm{SU}(d)}$ to achieve

$$W_{\otimes^n \mathrm{SU}(d)}(\theta,\phi) = \mathrm{Tr}\left[\hat{U}(\theta,\phi)\hat{\rho}\hat{U}^{\dagger}(\theta,\phi)\hat{\Pi}_{\otimes^n \mathrm{SU}(d)}\right]. \tag{24}$$

Following this convention, we can use the predefined functions in QuTiP [14] to compute the QPD for density matrices of arbitrary spin dimensions. Our analysis involves the normalization of the Husimi-Q QPD values such that the highest probability amplitude is unity. The computed Wigner function is kept as-is due to the physical significance of the values. A gallery of both functions for two-mode qudit states is displayed in Supplementary Figure 10.

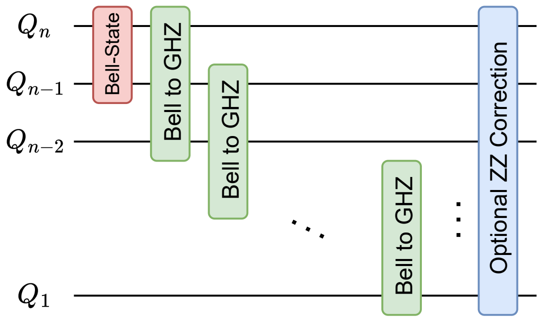

Supplementary Figure 11. **Bell-to-GHZ generalization**. A qudit circuit of arbitrary size that prepares the GHZ state with the terminating correction or some similar maximally entangling state without it. After initializing a Bell state, this circuit repeatedly applies the Bell-to-GHZ kernel building up entangling one qudit at a time.

Supplementary Note 8 – High-dimensional Quantum Circuit Synthesis

This note describes our approach for synthesizing high-dimensional state preparation circuits. Using this procedure, we developed the analytical solutions for preparing GHZ circuits of any size n and systems of any dimension d, using the reported two-photon transitions.

Two-qudit Bell state preparation

We begin by briefly describing our approach for the preparation of two-qudit Bell-states (Bell$_d$). Noting that for qudit-dimension d, we define a *Bell* state to be the pure and maximally entangled state given as

$$|\mathrm{Bell}_d\rangle = \frac{1}{\sqrt{d}}\sum_{k=0}^{d-1} e^{i\phi_k}|kk\rangle \tag{25}$$

where we allow $\phi_k \in [0,2\pi]$ to be an arbitrary phase on each state $|kk\rangle$ for $k = \{0,1,\ldots,d-1\}$.

The Bell$_d$ states can be compactly generated from the vacuum (ground) state $|00\rangle$ with selective two-photon drives between $|k,k\rangle \leftrightarrow |k+1,k+1\rangle$. In particular, a Bell$_2$ state is realized via a single π rotation of the $|00\rangle \leftrightarrow |11\rangle$ transition. A Bell$_3$ state is formed by a $2\pi/3$ rotation between $|00\rangle \leftrightarrow |11\rangle$ followed by a π rotation between $|11\rangle \leftrightarrow |22\rangle$, where the state progresses (up to arbitrary phases ϕ_k) as $|00\rangle \rightarrow \sqrt{\frac{1}{3}}|00\rangle + \sqrt{\frac{2}{3}}|11\rangle \rightarrow \frac{1}{\sqrt{3}}(|00\rangle + |11\rangle + |22\rangle)$. Similarly, Bell$_4$ requires a $3\pi/4$ rotation between $|00\rangle \leftrightarrow |11\rangle$, followed by a $2\pi/3$ rotation between $|11\rangle \leftrightarrow |22\rangle$, and finally a π rotation between $|22\rangle \leftrightarrow |33\rangle$. The experimental results for the tomographically reconstructed Bell$_d$ states for $d = \{2,3,4\}$ can be observed in Fig. 4 in the main text.

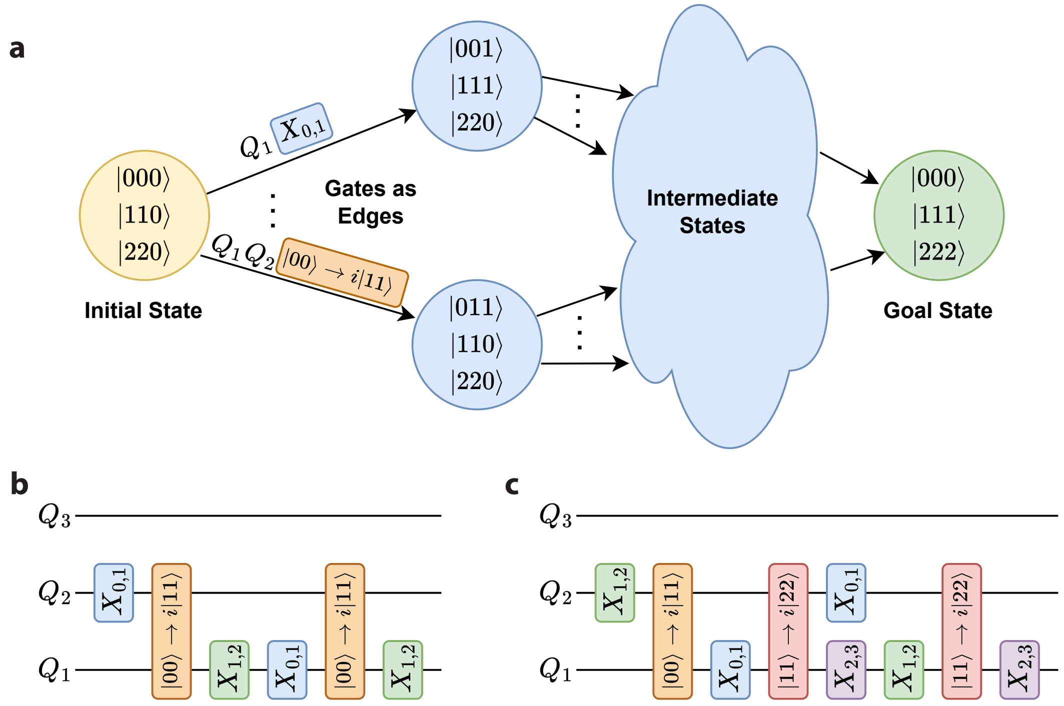

Supplementary Figure 12. **Algorithm for circuit compilation**. (**a**) Algorithm workflow: the quantum state preparation problem can be simplified to a shortest path search when no parameterized gates are necessary. Each state becomes a node. Applying gates transitions one node to another, linking them in the graph. We attribute a cost to each gate and utilize Dijkstra's algorithm to search for the best path to a target state from some initial state. (**b**) Kernel for generating qutrit ($d = 3$) GHZ states. (**c**) Kernel for generating ququart ($d = 4$) GHZ states.

Kernel cycling: Modular approach to state preparation

Next, we decompose the problem of GHZ state preparation into many applications of a three-qudit kernel. We designed this kernel to transition a Bell$_d$ state to a GHZ$_d$ state on three qudits, allowing us to cycle the application to build a GHZ state preparation circuit of any size, as shown in Supplementary Figure 11. We start by preparing a qudit Bell$_d$ state on the first pair of qudits as outlined in the previous section. Then, on the first three qudits, we apply this transition defined by:

$$\sum_{k<d} |k, k, 0\rangle \to \sum_{k<d} |k, k, k\rangle. \tag{26}$$

Afterward, we can cycle out the first qudit and cycle in the next one, resetting the system to the Bell$_d$ state. It is important to note that we are ignoring leading phases throughout this process. These can be corrected at the end of the circuit if desired.

Kernel synthesis: Dijkstra's algorithm

We desire a synthesized circuit that is composed without the use of Z rotations to minimize error accumulation. This restriction rules out the usage of state-of-the-art numerical synthesizers [31], as they require parameterized gates to be effective.

Instead, we mapped our synthesis problem to a shortest path search by embedding the possible circuits as paths in a directed graph, as shown in Supplementary Figure 12**a**. Here, nodes are unparameterized states, and edges are gates that transition one state to another.

We utilize Dijkstra's algorithm to search for the shortest path to a GHZ state given the bell state starting node. We assign a cost to each edge such that the graph has no negative weights, enabling Dijkstra's algorithm to find the shortest path quickly.

Preparation of GHZ states

After applying the previous method to find solutions for qutrit, ququart, and ququint GHZ kernels, we discovered a pattern. In Supplementary Figure 12**b,c**, we display the qutrit and ququart kernels. Here, we constructively prove that one can build a d-dimensional Bell to GHZ qudit state transition in $d-1$ two-qudit gates. We start with the qudit Bell state, $\sum_{k<d} |k, k, 0\rangle$. In the first step, we simply apply an X gate to the $d-3$ and $d-2$ subspaces in the middle qudit, giving us:

$$\sum_{k<(d-3)} |k,k,0\rangle + |(d-3),(d-2),0\rangle + |(d-2),(d-3),0\rangle + |(d-1),(d-1),0\rangle\,. \tag{27}$$

Next, for all $l < d-3$, we apply a two-photon transition, $|l,l\rangle \rightarrow i\,|(l+1),(l+1)\rangle$ to the last two qudits, followed by an X gate to the last qudit in the l and $l+1$ subspaces,

$$\sum_{k<(d-2)} |k,(k+1),k\rangle + |(d-2),(d-3),(d-3)\rangle + |(d-1),(d-1),(d-3)\rangle\,. \tag{28}$$

Then, we apply another two-qudit transition $|(d-3),(d-3)\rangle \rightarrow i\,|(d-2),(d-2)\rangle$ on the last two qudits, followed by a sequence of X gates on the middle qudit. Counting l up from zero to $d-3$, we apply an X gate in the l and $l+1$ subspaces:

$$\sum_{k<(d-3)} |k,k,k\rangle + |(d-3),(d-2),(d-3)\rangle + |(d-2),(d-2),(d-2)\rangle + |(d-1),(d-1),(d-3)\rangle\,. \tag{29}$$

Finally, we apply two X gates on the last qudit in subspaces $(d-2,d-1)$ and $(d-3,d-2)$, followed by a transition $|(d-3),(d-3)\rangle \rightarrow i\,|(d-2),(d-2)\rangle$. One more X gate on the rightmost qudit in the $(d-2,d-1)$ subspaces completes the algorithm to reach $\sum_{k<d} |k,k,k\rangle$.

This construction requires $d-1$ two-photon transitions. Furthermore, an n-qudit circuit will require the application of $n-2$ of these kernels alongside a bell-state preparation. The bell state preparation requires $d-1$ two-qudit gates, implying the total number of transitions for a GHZ circuit is $(n-1)(d-1)$, making it bilinear in qudit count and radix.

Preparation of non-local Bell states

We describe below the method by which we leverage two-photon qudit swaps to transform from a local to non-local Bell$_3$ state on the $Q_1-Q_2-Q_3$ system as in Supplementary Figure 12. We start by preparing our two-qudits in a Bell$_3$ state with the neighboring qudit in the ground state,

$$|\psi\rangle = \frac{1}{\sqrt{3}}(|000\rangle + |110\rangle + |220\rangle). \tag{30}$$

We then apply a local rotation to swap Q_3 from $|0\rangle$ to $|2\rangle$,

$$|\psi\rangle = \frac{1}{\sqrt{3}}(|002\rangle + |110\rangle + |222\rangle). \tag{31}$$

From here, we apply two sequential two-photon swaps, fully swapping the $Q_2 - Q_3$ $|22\rangle$ state to $|00\rangle$,

$$|\psi\rangle = \frac{1}{\sqrt{3}}(|002\rangle + |110\rangle + |200\rangle). \tag{32}$$

We proceed by again applying $|1\rangle \leftrightarrow |2\rangle$ local rotation on Q_3 yielding,

$$|\psi\rangle = \frac{1}{\sqrt{3}}(|002\rangle + |111\rangle + |201\rangle). \tag{33}$$

We next perform a final $Q_2 - Q_3$ $|11\rangle \leftrightarrow |00\rangle$ swap, generating

$$|\psi\rangle = \frac{1}{\sqrt{3}}(|002\rangle + |100\rangle + |201\rangle). \tag{34}$$

And finally to generate our non-local Bell$_3$ state, we apply $|1\rangle \leftrightarrow |2\rangle$ and $|0\rangle \leftrightarrow |1\rangle$ local rotations to Q_3,

$$|\psi\rangle = \frac{1}{\sqrt{3}}(|000\rangle + |101\rangle + |202\rangle). \tag{35}$$

In total, transforming from a local to non-local Bell$_3$ state requires 4 full two-photon swap operations, as well as a number of local rotation gates. This circuit complexity largely accounts for the sharp drop in state fidelity for increasingly non-local Bell$_3$ states in Fig. 5 in the main text. Notably, control errors leading to residual entanglement involving the intermediate qudits will manifest as a loss of purity, because the tomography on the two non-nearest-neighbor qudits traces out the intermediate qudits.

[1] J. Kreikebaum, K. O'Brien, A. Morvan, and I. Siddiqi, Improving wafer-scale josephson junction resistance variation in superconducting quantum coherent circuits, Superconductor Science and Technology **33**, 06LT02 (2020).

[2] D. C. McKay, C. J. Wood, S. Sheldon, J. M. Chow, and J. M. Gambetta, Efficient Z gates for quantum computing, Phys. Rev. A **96**, 022330 (2017).

[3] H. de Guise, O. Di Matteo, and L. L. Sánchez-Soto, Simple factorization of unitary transformations, Phys. Rev. A **97**, 022328 (2018).

[4] E. Knill, D. Leibfried, R. Reichle, J. Britton, R. B. Blakestad, J. D. Jost, C. Langer, R. Ozeri, S. Seidelin, and D. J. Wineland, Randomized benchmarking of quantum gates, Phys. Rev. A **77**, 012307 (2008).

[5] A. Morvan, V. V. Ramasesh, M. S. Blok, J. M. Kreikebaum, K. O'Brien, L. Chen, B. K. Mitchell, R. K. Naik, D. I. Santiago, and I. Siddiqi, Qutrit randomized benchmarking, Phys. Rev. Lett. **126**, 210504 (2021).

[6] R. Sarkar and T. J. Yoder, The qudit pauli group: non-commuting pairs, non-commuting sets, and structure theorems, Quantum **8**, 1307 (2024).

[7] R. Bianchetti, S. Filipp, M. Baur, J. M. Fink, C. Lang, L. Steffen, M. Boissonneault, A. Blais, and A. Wallraff, Control and tomography of a three level superconducting artificial atom, Phys. Rev. Lett. **105**, 223601 (2010).

[8] K. Mølmer and A. Sørensen, Multiparticle entanglement of hot trapped ions, Phys. Rev. Lett. **82**, 1835 (1999).

[9] K. N. Nesterov, Q. Ficheux, V. E. Manucharyan, and M. G. Vavilov, Proposal for entangling gates on fluxonium qubits via a two-photon transition, PRX Quantum **2**, 020345 (2021).

[10] L. B. Nguyen, Y. Kim, A. Hashim, N. Goss, B. Marinelli, B. Bhandari, D. Das, R. K. Naik, J. M. Kreikebaum, A. N. Jordan, *et al.*, Programmable heisenberg interactions between floquet qubits, Nature Physics , 240 (2024).

[11] S. Poletto, J. M. Gambetta, S. T. Merkel, J. A. Smolin, J. M. Chow, A. D. Córcoles, G. A. Keefe, M. B. Rothwell, J. R. Rozen, D. W. Abraham, C. Rigetti, and M. Steffen, Entanglement of two superconducting qubits in a waveguide cavity via monochromatic two-photon excitation, Phys. Rev. Lett. **109**, 240505 (2012).

[12] K. X. Wei, I. Lauer, E. Pritchett, W. Shanks, D. C. McKay, and A. Javadi-Abhari, Native two-qubit gates in fixed-coupling, fixed-frequency transmons beyond cross-resonance interaction (2023), arXiv:2310.12146 [quant-ph].

[13] L. B. Nguyen *et al.*, Blueprint for a high-performance fluxonium quantum processor, PRX Quantum **3**, 037001 (2022).

[14] J. Johansson, P. Nation, and F. Nori, QuTiP: An open-source python framework for the dynamics of open quantum systems, Computer Physics Communications **183**, 1760 (2012).

[15] F. Arute, K. Arya, R. Babbush, D. Bacon, J. C. Bardin, R. Barends, R. Biswas, S. Boixo, F. G. Brandao, D. A. Buell, *et al.*, Quantum supremacy using a programmable superconducting processor, Nature **574**, 505 (2019).

[16] P. Liu, R. Wang, J.-N. Zhang, Y. Zhang, X. Cai, H. Xu, Z. Li, J. Han, X. Li, G. Xue, W. Liu, L. You, Y. Jin, and H. Yu, Performing SU(d) operations and rudimentary algorithms in a superconducting transmon qudit for $d = 3$ and $d = 4$, Phys. Rev. X **13**, 021028 (2023).

[17] S. Cao, M. Bakr, G. Campanaro, S. D. Fasciati, J. Wills, D. Lall, B. Shteynas, V. Chidambaram, I. Rungger, and P. Leek, Emulating two qubits with a four-level transmon qudit for variational quantum algorithms (2023), arXiv:2303.04796 [quant-ph].

[18] N. Goss, S. Ferracin, A. Hashim, A. Carignan-Dugas, J. M. Kreikebaum, R. K. Naik, D. I. Santiago, and I. Siddiqi, Extending the computational reach of a superconducting qutrit processor (2023), arXiv:2305.16507 [quant-ph].

[19] M. S. Blok, V. V. Ramasesh, T. Schuster, K. O'Brien, J. M. Kreikebaum, D. Dahlen, A. Morvan, B. Yoshida, N. Y. Yao, and I. Siddiqi, Quantum information scrambling on a superconducting qutrit processor, Phys. Rev. X **11**, 021010 (2021).

[20] A. Cervera-Lierta, M. Krenn, A. Aspuru-Guzik, and A. Galda, Experimental high-dimensional Greenberger-Horne-Zeilinger entanglement with superconducting transmon qutrits, Phys. Rev. Appl. **17**, 024062 (2022).

[21] G. Silva, S. Glancy, and H. M. Vasconcelos, Investigating bias in maximum-likelihood quantum-state tomography, Physical Review A **95**, 022107 (2017).

[22] J. Bavaresco, N. Herrera Valencia, C. Klöckl, M. Pivoluska, P. Erker, N. Friis, M. Malik, and M. Huber, Measurements in two bases are sufficient for certifying high-dimensional entanglement, Nature Physics **14**, 1032 (2018).

[23] R. McWeeny, Some recent advances in density matrix theory, Reviews of Modern Physics **32**, 335 (1960).

[24] F. Yan, P. Krantz, Y. Sung, M. Kjaergaard, D. L. Campbell, T. P. Orlando, S. Gustavsson, and W. D. Oliver, Tunable coupling scheme for implementing high-fidelity two-qubit gates, Phys. Rev. Appl. **10**, 054062 (2018).

[25] V. Tripathi, H. Chen, M. Khezri, K.-W. Yip, E. Levenson-Falk, and D. A. Lidar, Suppression of crosstalk in superconducting qubits using dynamical decoupling, Phys. Rev. Appl. **18**, 024068 (2022).

[26] D. Rosenberg, D. Kim, R. Das, D. Yost, S. Gustavsson, D. Hover, P. Krantz, A. Melville, L. Racz, G. O. Samach, S. J. Weber, F. Yan, J. L. Yoder, A. J. Kerman, and W. D. Oliver, 3d integrated superconducting qubits, npj Quantum Information **3**, 42 (2017).

[27] F. T. Arecchi, E. Courtens, R. Gilmore, and H. Thomas, Atomic coherent states in quantum optics, Phys. Rev. A **6**, 2211 (1972).

[28] M. Kitagawa and M. Ueda, Squeezed spin states, Phys. Rev. A **47**, 5138 (1993).

[29] C. M. Caves and B. L. Schumaker, New formalism for two-photon quantum optics. I. Quadrature phases and squeezed states, Phys. Rev. A **31**, 3068 (1985).

[30] R. P. Rundle, P. W. Mills, T. Tilma, J. H. Samson, and M. J. Everitt, Simple procedure for phase-space measurement and entanglement validation, Phys. Rev. A **96**, 022117 (2017).

[31] E. Younis, C. C. Iancu, W. Lavrijsen, M. Davis, and E. Smith, Berkeley quantum synthesis toolkit (BQSKit) v1, Computer Software (2021).
